# Acute Facial Nerve Palsy in Facial Nerve Schwannoma Following COVID-19 Infection

**DOI:** 10.7759/cureus.36764

**Published:** 2023-03-27

**Authors:** Mohd Syafeeq Mohd Ridzam, Asma Abdullah

**Affiliations:** 1 Department of Otorhinolaryngology-Head and Neck Surgery, Hospital Canselor Tuanku Muhriz, Universiti Kebangsaan Malaysia Medical Centre, Kuala Lumpur, MYS

**Keywords:** facial nerve paralysis, acute facial nerve palsy, facial nerve neuroma, covid-19, facial nerve schwannoma

## Abstract

Lower motor neuron facial nerve palsy (FNP) has many causes. Bell’s palsy is by far the commonest cause. Among other causes include infective and neoplastic causes. While FNP caused by facial nerve schwannoma (FNS); a benign neoplastic condition of the facial nerve is slowly progressing, infective causes mainly viral origins present with acute FNP. We present a young female who complained of an acute onset of FNP on day five of her COVID-19 infection. She initially presented with symptoms suggestive of ear infection, and subsequent magnetic resonance imaging (MRI) showed evidence of FNS, which she was subjected to surgery later at our center. This rare acute incidence of FNP in schwannoma might be triggered by the COVID-19 infection and demonstrates the role of imaging in finding the cause of FNP.

## Introduction

The common causes of lower motor neuron facial nerve palsy (FNP) include trauma, infection, iatrogenic, tumor, and idiopathic [1]. Bell’s palsy, a diagnosis of exclusion, is the commonest cause of this limiting condition, with an incidence of 10-40 cases per 100,000 population each year [1,2]. The most common tumor of the facial nerve, facial nerve schwannoma (FNS) accounts for less than 5% of all causes of FNP [3]. This is not surprising given the rarity of this condition. FNS commonly affects the middle-aged population, with a mean age of between 45-47 years old [4-6]. This condition, though benign, can affect a patient’s daily activities and can be debilitating. While some patients have intact facial nerve function, mostly will demonstrate slow-progressing FNP.

The recent SARS-CoV-2 virus, which is responsible for the devastating global COVID-19 pandemic, has recently shown the possibility of neurogenic complications. One of which is the involvement of facial nerve palsy or paresis [7,8]. A systematic review by Namavarian et al. described occurrences of COVID-19 that presented with FNP as an isolated symptom or in combination with other systemic symptoms. Though rare, fortunately, most FNPs are short-lived like other infective causes. FNP inflicted by COVID-19 recovers earlier but with a higher proportion of bilateral FNP in comparison to Bell’s palsy [7].

## Case presentation

A healthy young lady in her 30s with no past medical history was referred to us for a sudden unilateral FNP on day five of COVID-19 infection. The diagnosis was done by reverse transcriptase-polymerase chain reaction (RT-PCR) test. She did not require admission or any oxygen supplement. She had typical influenza-like symptoms for a week, followed by left otorrhea and otalgia with associated sudden onset left facial weakness. She had neither tinnitus, vertigo, nor reduced hearing. She was then being treated with antibiotic ear drops.

**Figure 1:**
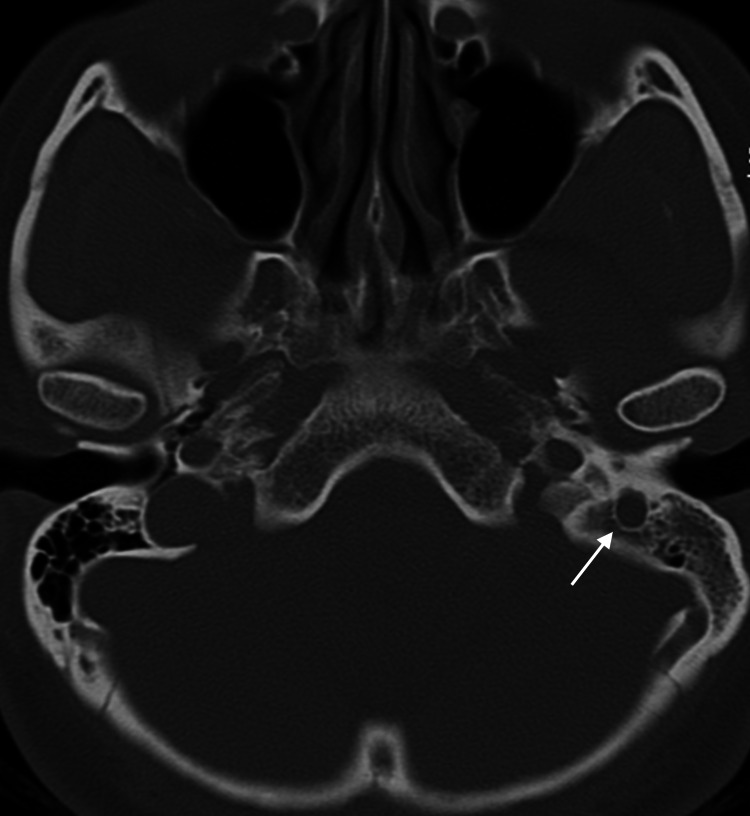
An axial view of high-resolution CT of the temporal bone. Arrow shows a widening of the stylomastoid foramen likely due to enlargement of the facial nerve.

**Figure 2:**
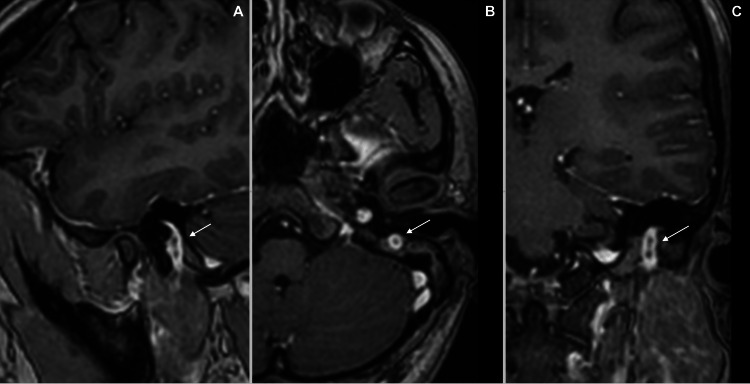
Gadolinium-enhanced T1-weighted image of MRI (internal acoustic meatus and brain). (A) Sagittal view, (B) axial view, and (C) coronal view. Arrow shows an enhancing left mastoid segment of the facial nerve causing the widening of the stylomastoid foramen. MRI: magnetic resonance imaging.

Our assessment revealed left FNP House-Brackmann (HB) grade III with an otoscopic examination showed left tympanosclerosis with healed perforation. The pure tone audiometry and tympanogram were normal. As the clinical findings were not suggestive of an acute infection, high-resolution computed tomography (HRCT) of the temporal was performed and reported as an enlarged stylomastoid foramen with likely enlargement of the seventh nerve (Figure 1). Magnetic resonance imaging (MRI) with gadolinium contrast of the internal acoustic meatus and brain was performed and revealed an enhancing left mastoid segment of the facial nerve, causing an enlarged stylomastoid foramen. Differential diagnosis includes neuroma and schwannoma (Figure 2). 

**Figure 3:**
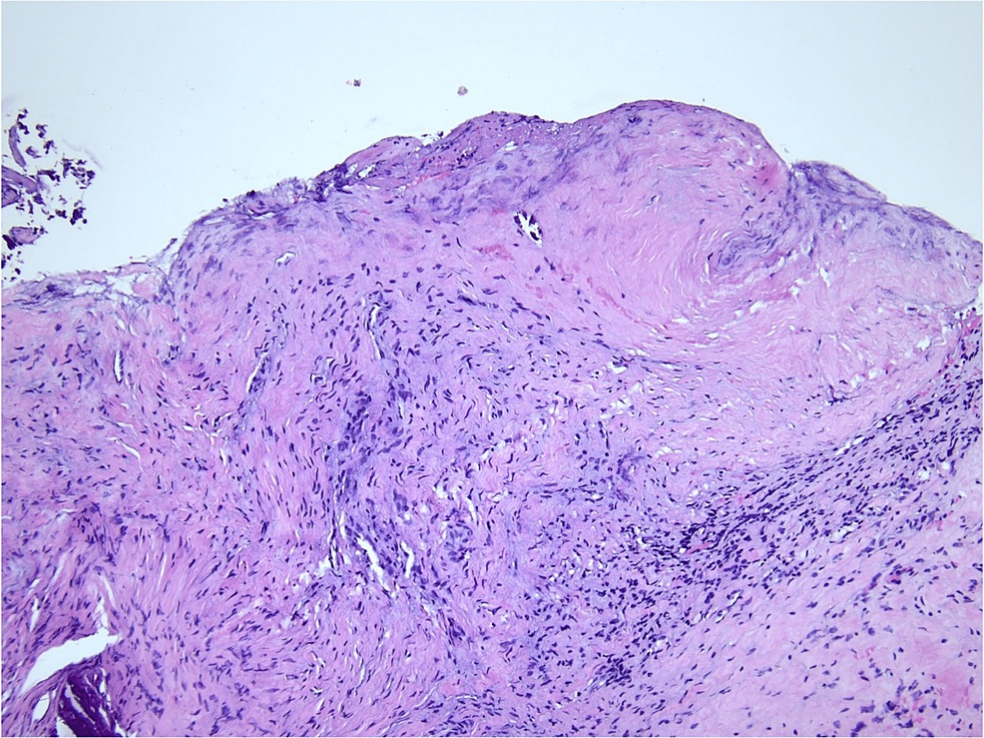
Tissue composed of relatively bland spindle-shaped cells arranged in loose fascicles. Hypercellular areas (Antoni A) were observed. No Verocay bodies were seen.

**Figure 4:**
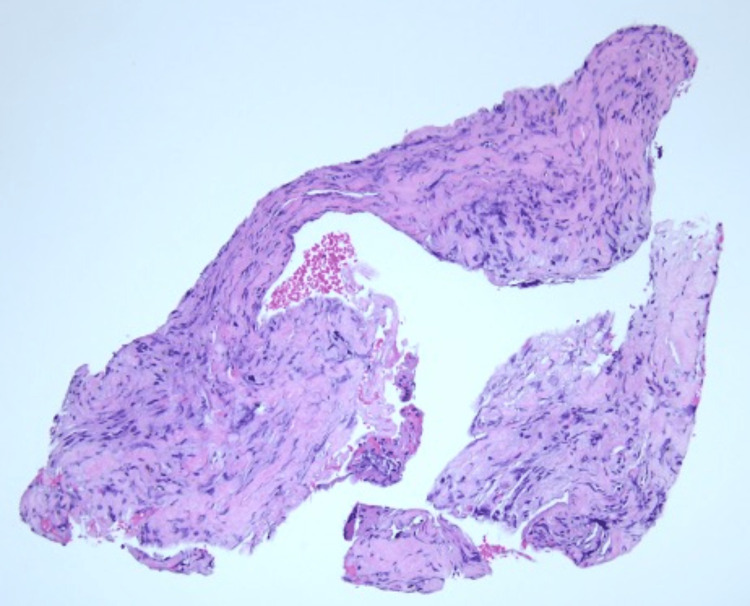
Tissue composed of relatively bland spindle-shaped cells arranged in loose fascicles. Hypocellular areas (Antoni B) were observed. No Verocay bodies were seen.

Facial asymmetry did not improve; hence, surgery was performed. She had a left cortical mastoidectomy, posterior tympanotomy, tumor excision, and neurolysis performed. Intraoperatively, a tumor was seen from the proximal to the distal portion of the mastoid segment of the left facial nerve. The tumor and perineurium were removed from the intact nerve, while neurolysis was carried out. Histopathological examination of the specimen was reported as FNS with evidence of the Antoni A and B patterns (Figures 3, 4). The immunostaining for S100 was positive, which confirmed the diagnosis. Six months postoperatively, her facial nerve function improved to HB grade II.

## Discussion

FNS is rare, accounting for less than 1% of all temporal bone tumors [9]. It originates from the Schwann cell, confines within a capsule and stays at the periphery of the facial nerve. The most common site affected is the intra-temporal part of the facial nerve, specifically the geniculate ganglion and labyrinthine segment. Most patients with FNS present with hearing loss, FNP, tinnitus, vertigo, and facial spasm [10]. Based on the literature, hearing loss and facial nerve palsy are the two most common symptoms complained by patients with FNS [5,10].

Some studies report FNP as the most common presentation for FNS [10], with HB II-III FNP at diagnosis. In contrast, other studies reported hearing loss as the commonest symptom, such as the study by Carlson et al., in which a lot of patients had intact facial nerve function at the time of diagnosis [5]. In these cases, the FNP is slow to progress taking three weeks or more and may relapse. This is partly due to the slow-progressing nature of the tumor; at a rate of 0.85-1.4 mm per year [6]. 

The combination of MRI and HRCT temporal is important to get the right diagnosis as they demonstrate the primary tumor of the facial nerve. However, HRCT temporal is better at depicting smooth osseous widening of the bony facial canal and the relationship of the facial canal to normal anatomic landmarks such as the ossicles [5,10]. On the other hand, MRI is more sensitive in demonstrating the schwannoma as well as differentiating other tumors such as hemangiomas.

Treatment for FNS includes watchful waiting, surgery, and stereotactic surgery. Surgery includes radical/subtotal resection, nerve decompression, and facial nerve grafting [6]. A lot of factors have to be considered to decide for treatment; the location and volume of the tumor, intracranial mass effect, preoperative nerve status, patient’s overall conditions, as well as patient’s preferences. High-grade FNP (HB≥III), especially with intracranial mass effect, is indicated for radical surgery, whereas those asymptomatic or with small tumors can be observed closely. Subtotal resection is best for the patient with slight functional deterioration with the tumor potentially affecting nearby structures [6]. The close proximity of the schwannoma to the otic capsule may expose the risk of hearing loss during resection; hence, surgery may be avoided in the only hearing ear. The approach taken for surgery is much dependent on the hearing status of the affected side as well as the contralateral side [11]. Recent treatment modalities, stereotactic surgery is best for the elderly, those who are not fit for surgery or recurrence after surgery. It slows tumor growth, spare hearing, and facial nerve function but its long-term effectiveness is yet to be proven [10]. Prognosis varies based on the duration, degree of preoperative FNP as well as the surgical technique performed.

Other more common cause of FNP is infection. Viral infection has been known to cause temporary facial nerve palsy, for instance, varicella zoster, human immunodeficiency virus (HIV), and mumps. Bacterial causes may include Lyme disease and Mycobacterium tuberculosis [8]. Recently, there were reports that patients with COVID-19 infection presented with acute FNP. A recent systematic review in 2021 by Namavarian et al. reported that patients with COVID-19 infection may have FNP, either associated with Guillain-Barré syndrome or as isolated symptoms [7]. Unlike other viral causes of FNP, the onset of FNP in COVID-19 infection is nine days on average (ranging between one and 20 days) as compared to other viral causes, which typically occur concurrently with other symptoms. It is hypothesized that there is viral dissemination to the central nervous system and cranial nerve, causing neuronal damage. Other theory blames the hypercoagulability state of the COVID-19 infection for causing vascular damage to the vasa nervorum. Autoimmune reactions causing nerve inflammation are also an alternative explanation [8].

In this case, the patient has an acute FNP on day five of the COVID-19 infection with symptoms suggestive of an ear infection. Given that the patient was asymptomatic prior to getting infected with COVID-19, we postulate that the COVID-19 infection may be the triggering factor for the FNP, hence the acute onset. It was not surprising that she had an intact facial nerve prior to COVID-19 as the tumor was at the mastoid segment of the facial nerve. According to Carlson et al., most FNS with FNP were those with tumors involving the restricted segments, the labyrinthine and tympanic segments. At these narrowed areas, neurocompression occurs, causing neural injury and ischemia [5]. In this case, COVID-19 viral dissemination may trigger neural injury to the susceptible swollen tumor segment causing FNP.

## Conclusions

FNS may cause FNP, which is slowly progressing and can be relapsing or permanent. The role of radiological imaging is paramount in finding the exact etiology of the FNP and subsequently determining the appropriate modality of treatment for the patient. Surgery, stereotactic surgery, or watchful waiting is the treatment options for symptomatic FNS. Though rare, COVID-19 infection as a cause of acute FNP should not be underestimated, while other more common infective causes should remain on top of the list. These infective causes are usually short-lived. 
